# Roles of Nicotinamide N-Methyltransferase in Obesity and Type 2 Diabetes

**DOI:** 10.1155/2021/9924314

**Published:** 2021-07-27

**Authors:** Jie-Ru Liu, Zhao-Hui Deng, Xiao-Juan Zhu, Yu-Rong Zeng, Xiang-Xiang Guan, Jiang-Hua Li

**Affiliations:** Key Lab of Aquatic Training Monitoring and Intervention of General Administration of Sport of China, Physical Education College, Jiangxi Normal University, Nanchang, 330022 Jiangxi, China

## Abstract

Type 2 diabetes (T2D) is thought to be a complication of metabolic syndrome caused by disorders of energy utilization and storage and characterized by insulin resistance or deficiency of insulin secretion. Though the mechanism linking obesity to the development of T2D is complex and unintelligible, it is known that abnormal lipid metabolism and adipose tissue accumulation possibly play important roles in this process. Recently, nicotinamide N-methyltransferase (NNMT) has been emerging as a new mechanism-of-action target in treating obesity and associated T2D. Evidence has shown that NNMT is associated with obesity and T2D. NNMT inhibition or *NNMT* knockdown significantly increases energy expenditure, reduces body weight and white adipose mass, improves insulin sensitivity, and normalizes glucose tolerance and fasting blood glucose levels. Additionally, trials of oligonucleotide therapeutics and experiments with some small-molecule NNMT inhibitors *in vitro* and in preclinical animal models have validated NNMT as a promising therapeutic target to prevent or treat obesity and associated T2D. However, the exact mechanisms underlying these phenomena are not yet fully understood and clinical trials targeting NNMT have not been reported until now. Therefore, more researches are necessary to reveal the acting mechanism of NNMT in obesity and T2D and to develop therapeutics targeting NNMT.

## 1. Introduction

Obesity is a predisposing factor for T2D, and the prevalence of obesity has significantly accelerated the rise of T2D in the past few decades. T2D is thought to be a complication of metabolic syndrome caused by disorders of energy utilization and storage [1] and characterized by insulin resistance and deficiency of insulin secretion [2]. The mechanism linking obesity and the development of T2D is complex and unintelligible, but it is known that abnormal lipid metabolism and adipose tissue accumulation possibly play important roles [3]. The increase in the circulatory free fatty acid (FFA) concentration in plasma and the elevation of lipid deposition in skeletal muscles caused by abnormal lipid metabolism are the important signs of insulin resistance and T2D [4]. Increased plasma FFAs reduce glucose uptake stimulated by insulin, but the decrease of lipid content improves insulin activities in skeletal muscles, adipocytes, and the livers [4, 5]. It was proposed that the elevated levels of FFAs in plasma causes insulin resistance by inhibiting glucose transport and/or phosphorylation activities [4]. Adipose tissues release numerous metabolic hormones, cytokines, and other mediators, such as glycerol, leptin, adiponectin, and nonesterified fatty acids (NEFAs) [6]. An increase in the NEFA level in plasma is observed in patients with obesity and T2D and is significantly associated with insulin resistance [6, 7]. Increased NEFAs in cells compete with glucose for substrate oxidation, thus resulting in the inhibition of pyruvate dehydrogenase, phosphofructokinase, and hexokinase II [8].

Although it is known that abnormal lipid metabolism and adipose tissue accumulation possibly play important roles in linking obesity and T2D, the actual mechanisms are still unclear because not all obese people are insulin resistant or at high risk of diabetes [9]. Recently, NNMT has been emerging as a new mechanism-of-action target for treatment of obesity and associated T2D [10]. This review will focus on the roles of NNMT in obesity and T2D, as it has been found that the expression of this methyltransferase is elevated in the white adipose tissue and liver of obese people and diabetic mice and that *NNMT* knockdown protects against diet-induced obesity and insulin resistance [11].

## 2. Physiological Function of NNMT and the Related Metabolic Pathways

**Figure 1:**
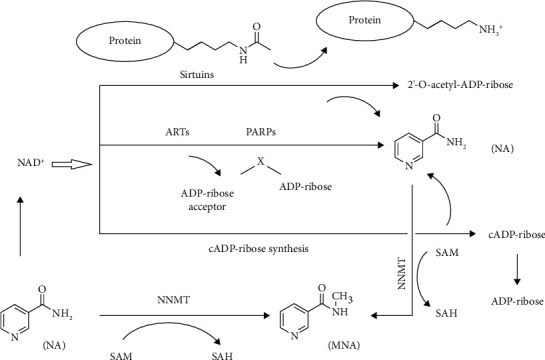
Physiological function of nicotinamide N-methyltransferase (NNMT) and the related metabolic pathways. NA: nicotinamide; MNA: 1-methylnicotinamide; SAM: S-adenosyl-methionine; SAH: S-adenosyl-homocysteine; NAD^+^: nicotinamide adenine dinucleotide; ARTs: ADP-ribosyl transferases; PARPs: poly-ADP-polymerases.

NNMT is a cytosolic enzyme that catalyzes NA methylation using S-adenosyl-methionine (SAM) as a methyl donor, while producing 1-methylnicotinamide (MNA) and S-adenosyl-homocysteine (SAH). As Figure 1 shows, NA is a precursor of NAD^+^ and also a catabolic product of NAD^+^. NAD^+^ is mainly consumed in three ways, in which NA is produced, and 4 enzymes, sirtuins, ADP-ribosyl transferases (ARTs), poly-ADP-polymerases (PARPs), and cADP-ribose synthases, are involved (Figure 1). Therefore, the methylation of NA is not only relevant to the abundance of free NAD^+^ but is also relevant to the activity of these NAD^+^-consuming enzymes, because as a metabolite of these NAD^+^-consuming reactions, NA naturally has inhibitive effects on these enzymes.

NAD^+^ is the key coenzyme for glycolysis and the tricarboxylic acid cycle and for the conversion of carbohydrates to lipids [12]. Sirtuins are a family of NAD^+^-dependent deacylases that have been shown to regulate a myriad of biological functions, ranging from cell growth to lifespan extension, including oxidative stress, DNA damage, glycolysis, gluconeogenesis, and lipogenesis [13–16]. ARTs, PARPs, and cADP-ribose synthases cleave NAD^+^ into NA and ADP-ribose (Figure 1). ADP-ribose is polymerized onto the nuclear proteins, including histones and transcription factors at DNA strand breaks, in DNA repair [17]. Normal concentrations of NA can prevent degradation of PARPs and allow DNA repair [18]. NA cannot be salvaged into NAD^+^ any longer if it is N-methylated by NNMT, so the methylation of NA impairs the abundance of free NAD^+^ and then affects the activities of these NAD^+^-consuming enzymes [19]. According to these findings, it was proposed that NNMT plays important roles in energy metabolism and in the development of a number of disorders, such as obesity, diabetes, aging, Parkinson's disease, and cancer [19]. This review article is based on works with a focus on the current research status quo of NNMT in the field of obesity and T2D.

## 3. Association between NNMT and Obesity

Early evidence of the association between NNMT and obesity was observed in metabolomic works, of which the results show that the MNA levels in human urine are positively correlated with the body mass index (BMI) [20, 21]. Recently, this association has been confirmed by many reports. For instance, the MNA levels in serum were found increased in the obese people [22] and the MNA levels in urine were found increased both in obese db/db mice and in obese Zucker rats [21]. These findings indicate that the activity of NNMT is increased in obese individuals. Evidence has shown that NNMT expression in white adipose tissue (WAT) is high in obesity-prone mice and low in obesity-resistant mice [23–25] and significantly correlates with the percent fat mass in diet-induced obese mice [26]. Lee et al. found that NNMT expression in adipocytes is increased in Pima Indians [27]. Kraus et al. [11] found that *NNMT* knockdown induced a 47% reduction in the relative fat mass of mice and proposed that NNMT in WATs and/or in livers may have a causative role in obesity. Brachs et al. [28] found that *NNMT* knockdown significantly reduced the body weight, fat mass, and insulin level of female mice fed a Western diet (fat 47% kcal, carbohydrate 34% kcal), as did *NNMT* knockout.

Since NNMT expression is directly determined by the *NNMT* gene, in our previous works, nineteen SNPs in the *NNMT* DNA sequence were selected as tagSNPs using Haploview software (Haploview 4.2) to observe the association between *NNMT* gene polymorphisms and obesity [29] and hyperlipidemia [30]. We found that the rs10891644 variation is significantly associated with obesity [29] and that the rs1941404 variation is significantly associated with hyperlipidemia in the Chinese population [30]. Recently, Bañales-Luna et al. [31] reported that Mexican subjects with rs694539 variation (genotype AA, recessive inherence model) in the *NNMT* gene sequence have lower BMI values.

## 4. Association between NNMT and T2D

Many reports have shown the association between NNMT and T2D. Quantitative trait locus mapping in mice demonstrated that *NNMT* plays a causative role in the development of T2D [32] and MNA levels were elevated in both the serum [22] and urine [21] of patients with T2D. These results indicate that NNMT activities are increased in the development of T2D. The more direct evidence is mainly from the following reports. Kannt et al. [33] demonstrated that NNMT expression is upregulated in WAT of humans with insulin resistance or T2D and that plasma MNA levels are significantly and positively correlated with NNMT expression in WAT and with the degree of insulin resistance. Kraus et al. [11] found that NNMT expression levels are elevated in WAT and the liver of the T2D mice and that *NNMT* knockdown improves the glucose tolerance and insulin sensitivity in the T2D mice. Subsequently, Hong et al. [14] confirmed the roles of NNMT in the development of T2D. *In vitro*, they found that *NNMT* knockdown significantly lowers hepatocyte glucose output (50%) and that *NNMT* overexpression significantly increases hepatocyte glucose output (1.4-fold) [14]. *In vivo*, they found that *NNMT* knockdown in the livers of C57BL6/J mice significantly lowers overnight fasting glucose levels [14].

In our previous works, the association between T2D and two SNPs in the *NNMT* gene sequence (rs694539 and rs1941404) was observed [34]. The rs694539 variation has shown the significant associations with many noninfectious chronic diseases (NCDs), such as hyperhomocysteinemia [35], nonalcoholic steatohepatitis [36], congenital heart diseases [37], migraine [38], bipolar disorder [39], schizophrenia [40], epilepsy [41], and abdominal aortic diseases [42], and the rs1941404 variation has been reported to be associated with hyperlipidemia [30]. The results show that the rs1941404 variation is significantly associated with T2D [34].

## 5. Potential Mechanisms Linking NNMT and T2D

T2D is thought to be a complication of metabolic syndrome caused by an underlying disorder of energy metabolism and characterized by impaired glucose tolerance or elevated fasting blood glucose [43]. In this section, we will focus on the roles of NNMT in regulating energy metabolism and glucose metabolism.

### 5.1. Roles of NNMT in Energy Metabolism and the Related Mechanisms

As a precursor of NAD^+^, NA methylation plays important roles in regulating energy metabolism because methylated NA is no longer able to be salvaged into NAD^+^ [16]. NAD^+^ is a key coenzyme for fuel oxidation and for the conversion of carbohydrates to lipids [44]. The competition between NA methylation and NAD^+^ salvage indicates that NNMT might limit fuel oxidation and promote fat storage, because if the activity or expression of NNMT is high, then, NA might not be salvageable, thus limiting the NAD^+^-dependent processes [16].

In our previous works, we found that NNMT genetic variants in the Chinese population are significantly associated with resting energy expenditure [30] and maximal oxygen uptake [45]. Recently, Bañales-Luna et al. [31] reported that NNMT genetic variants in Mexican subjects are also significantly associated with resting energy expenditure. The further roles of NNMT in regulating energy metabolism and the related mechanism were mainly from Kraus et al. [11]. *In vitro*, they found that *NNMT* knockdown and NNMT inhibition by MNA both significantly increase the oxygen consumption in adipocytes. However, *NNMT* overexpression significantly decreases the oxygen consumption in adipocytes [11]. *In vivo*, they found that *NNMT* knockdown mice had higher energy expenditures than the control mice with similar body weights [11]. Regarding the mechanism by which NNMT regulates energy expenditure, they proposed that SAM and NAD^+^ are involved because SAM and NAD^+^ are modulated in the process of NA methylation by NNMT [11]. NAD^+^ is a key cofactor linking cellular redox states to energy metabolism [46]. SAM provides propylamine for the biosynthesis of polyamine and donates a methyl group for the methylation of histone [47]. Activation of polyamine flux causes an increase in energy expenditure by catalyzing polyamine acetylation to produce acetylpolyamines using acetyl-coA as a substrate [48]. NNMT inhibition increases the levels of SAM and NAD^+^ and upregulates the activation of polyamine flux, resulting in an increase in energy expenditure [11].

### 5.2. Roles of NNMT in Glucose Metabolism and the Related Mechanisms

As mentioned above, T2D is characterized by impaired glucose tolerance or elevated fasting blood glucose [43]. In our previous works, a SNP (rs1941404) in the *NNMT* gene sequence was found significantly associated with impaired glucose tolerance and elevated fasting blood glucose [34]. The further roles of NNMT in regulating glucose metabolism and the related mechanisms are mainly from Kraus et al. [11] and Hong et al. [14].

Kraus et al. [11] reported that *NNMT* knockdown improves glucose tolerance and insulin sensitivity in diet-induced obese mice. Brachs et al. [28] confirmed these findings in their studies. They found that *NNMT* knockdown reduced the insulin level and improved glucose tolerance in female mice fed with a Western diet and that *NNMT* knockout strongly improved insulin sensitivity in male mice fed with a high-fat diet [28]. Hong et al. [14] initially tested the roles of NNMT in gluconeogenesis and found that *NNMT* knockdown decreases hepatocyte glucose output, glucose-6-phosphatase catalytic (G6pc) expression, and phosphoenolpyruvate carboxykinase 1 cytosolic (*Pck1*) expression in primary hepatocytes. On the contrary, *NNMT* overexpression increases hepatocyte glucose output, *G6pc* expression, and *Pck1* expression in primary hepatocytes. *In vivo*, Hong et al. [14] found that mice with *NNMT* knockdown have lower levels of fasting glucose and lower pyruvate conversion to glucose compared to control mice. These findings suggest that NNMT is a positive modulator of hepatocyte gluconeogenesis.

Regarding the mechanism by which NNMT regulates glucose metabolism, Hong et al. [14] proposed that the regulatory effect of NNMT is mediated by its product MNA and that Sirt1 is necessary in this regulatory process. First, they found that glucose production, *G6pc* expression, and *Pck1* expression were significantly lower in *NNMT*-overexpressing hepatocytes with Sirt1 inhibition than in *NNMT*-overexpressing hepatocytes without Sirt1 inhibition and that Sirt1 overexpression rescued the suppression of *G6pc* expression and *Pck1* expression induced by *NNMT* knockdown [14]. These results indicate that Sirt1 is necessary to mediate the regulatory effect of NNMT on glucose metabolism. Second, they found that Sirt1 protein expression is significantly correlated with *NNMT* expression in hepatocytes [14]. *In vitro*, Sirt1 protein expression was significantly increased in primary hepatocytes by *NNMT* overexpression and was significantly decreased in primary hepatocytes by *NNMT* knockdown [14]. *In vivo*, Sirt1 protein expression was also significantly decreased in the livers of mice by *NNMT* knockdown [14]. Third, they found that the enzymatic activity of NNMT is necessary for the increase in Sirt1 protein expression and a product of NNMT (MNA) plays a key role [14]. Similar to *NNMT* overexpression, MNA-treated hepatocytes show a dose-dependent increase in glucose production, Sirt1 protein expression, *G6pc* mRNA expression, and *Pck1* mRNA expression compared to controls and these changes caused by MNA treatment can be abolished by Sirt1 knockdown [14]. These findings suggest that both NNMT and its product MNA increase Sirt1 protein expression, while Sirt1 is needed for the regulatory effects of NNMT and MNA on glucose metabolism [14].

## 6. NNMT as a Therapeutic Target

Evidence has shown that *NNMT* knockdown leads to an increase in energy expenditure in adipocytes [11] and a decrease in glucose output in hepatocytes [14]. Moreover, in animals, *NNMT* knockdown improves insulin sensitivity, overnight fasting glucose levels, and glucose tolerance and reduces the relative fat mass [11, 14]. These findings render NNMT an attractive target in developing oligonucleotide drugs to prevent or treat obesity and T2D. However, to date, oligonucleotide therapeutics are still facing some technological obstacles, such as how to achieve efficient oligonucleotide delivery to target organs or tissues, how to overcome off-target interactions [49–52], and how to address the sequence- and chemistry-dependent toxicity and saturation of endogenous RNA processing pathways [53, 54]. Therefore, although some oligonucleotide products targeting NNMT have shown effectiveness *in vitro* and in animal experiments, to date, no oligonucleotide products targeting NNMT have been used in clinical trials. However, it is noteworthy that ten oligonucleotide drugs have been officially approved by FDA as of January 2020 [55], which means that the technological obstacles for oligonucleotide therapeutics are being solved step by step and the development of oligonucleotide drugs targeting NNMT is still a promising method for the prevention or treatment of obesity and related T2D in the future.

In addition to oligonucleotide therapeutics, some small-molecule NNMT inhibitors have been reported recently [56–59] and several have shown effectiveness in the prevention or treatment of obesity and T2D *in vitro* [60, 61] or in preclinical animal models [10, 58, 62], validating NNMT as a pharmacological drug target.

MNA, as the product of the NNMT enzymatic reaction, is widely used as an NNMT inhibitor in research on biological function of NNMT. *In vitro*, MNA has shown significant effects in increasing energy expenditure and modulating glucose metabolism [11, 14], but *in vivo*, its effects are controversial. In a report from Przyborowski et al. [63], after 4 weeks of MNA administration (100 mg/kg), no significant differences were found between MNA-treated and untreated diabetic db/db mice with regard to the levels of fasting blood glucose and HbA1c and the body weights of the MNA-treated db/db mice did not decrease but tended to increase compared to those of the untreated db/db mice. Hong et al. [14] also reported that MNA supplementation has no effects on blocking body weight gain. As a product of the NNMT enzymatic reaction, MNA is a natural inhibitor of NNMT. Swaminathan et al. [64] demonstrated the ternary complex X-ray crystal structures of NNMT in bound form with MNA, which indicates that MNA can bind to the active site of NNMT, thereby inhibiting NA binding and inhibiting the activities of NNMT. Why is the effect of MNA treatment *in vivo* not as good as it should be in the control of fasting blood glucose, HbA1c, and body weight? There are 2 factors that might explain this phenomenon. First, MNA is not stable in *in vivo*. It can be further oxidized to N-methyl-2-pyridone-5-carboxamide (2PY) and N-methyl-4-pyridone-3-carboxamide (4PY) *in vivo* and is easily excreted in urine in the forms of MNA, 2PY, and 4PY (in humans, the major metabolite of MNA is 2PY, and in rodents, the major metabolite of MNA is 4PY) [65–67]. Second, the membrane permeability of MNA is poor. Membrane permeability is crucial for a drug to obtain desirable treatment effects. According to the report from Neelakantan et al. [10], MNA exhibited no passive permeability in parallel artificial membrane permeability assay (PAMPA), which might partly explain the undesirable treatment effects of MNA in the control of body weight and blood glucose homeostasis.

Neelakantan et al. [10] found some 1-methylquinolinium scaffolds modified with primary amine substitutions displaying both high passive membrane diffusion in PAMPA and high active transport membrane permeability in a bidirectional permeability assay with Caco-2 cells, and one of them (5-amino-1-methylquinolinium (5-amino-1MQ)) has significant effectiveness against diet-induced obesity. Importantly, 5-amino-1MQ has high selectivity and does not inhibit the related SAM-dependent methyltransferases or enzymes in NAD^+^ salvage pathways. *In vitro*, 5-amino-1MQ significantly reduced intracellular MNA, increased intracellular NAD^+^, and suppressed lipogenesis in adipocytes. *In vivo*, 5-amino-1MQ-treated mice had significantly reduced body weights, white adipose masses, and adipocyte sizes. Notably, 5-amino-1MQ treatment did not show a significant impact on the food intake and any observable adverse effects. These results suggest that 5-amino-1MQ is a potent small-molecule NNMT inhibitor that reverses diet-induced obesity and related T2D.

In addition to 5-amino-1MQ, JBSNF-000088 is another NNMT inhibitor that is promising for treatment of obesity and associated T2D. JBSNF-000088, a small-molecule analog of NA, is able to be bound to NNMT and to be methylated in the NNMT structure, with SAM being correspondingly demethylated to SAH [58]. Therefore, the methylation of NA catalyzed by NNMT is inhibited, and the product, MNA, is reduced. In animal models of metabolic disease, JBSNF-000088 treatment reduced MNA levels and drove insulin sensitization, glucose modulation, and body weight reduction. In diet-induced obese mice, JBSNF-000088 treatment reduced body weights, improved insulin sensitivity and normalized glucose tolerance [58].

## 7. Implications and Recommendations for Future Studies

As summarized in this review, NNMT plays important roles in obesity and T2D and is an attractive therapeutic target to prevent or treat obesity and associated T2D. Although the current evidence is very promising, there are still some knowledge gaps that should be addressed to further clarify the exact mechanisms linking NNMT to obesity and T2D and to develop clinical drugs targeting NNMT.

Usually, increased energy expenditure is used to explain the protective effects of NNMT inhibition or *NNMT* knockdown against diet-induced obesity. NNMT catalyzes the methylation of NA, which is a precursor of NAD^+^. If NNMT expression or activity is high, then, NA might not be salvageable to form NAD^+^. The competition between NA methylation and NAD^+^ salvage indicates that NNMT might limit fuel oxidation and promote fat storage because NAD^+^ is necessary for fuel oxidation [16]. Kraus et al. [11] observed that *NNMT* knockdown increased both NAD^+^ levels and oxygen consumption in WAT. However, in the liver, *NNMT* overexpression did not change the levels of NAD^+^ [11, 14] but did depress oxygen consumption [11]. Therefore, the depression of energy expenditure caused by *NNMT* overexpression cannot be explained solely by the competition between NNMT and NAD^+^ salvaging. In addition, it seems that NNMT plays different roles in aerobic and anaerobic energy metabolism. If the depressed oxygen consumption caused by *NNMT* overexpression indicates the depression of aerobic metabolism, the regulatory effect of NNMT on anaerobic metabolism might be reversed. Generally, NNMT activity or expression is significantly elevated in cancers, such as brain [68], lung [69], liver [70], kidney [71, 72], bladder [73], stomach [74, 75], pancreas [76], colon [77], and oral [78, 79] cancer but cancer cells usually have a significantly higher anaerobic metabolism rate than normal cells. In one of our previous studies [80], we found that the levels of NNMT expression in extensor digitorum longus muscles (a typical type of fast-twitch muscle relying on anaerobic metabolism to produce power) are higher than those in the soleus muscle (a typical type of slow-twitch muscle relying on aerobic metabolism to produce power) and that anaerobic exercise is more effective in increasing NNMT expression in skeletal muscles compared to aerobic exercise. Recently, we inhibited NNMT in rats with MNA and found that NNMT inhibition impaired performance of the rats in anaerobic endurance exercise. These phenomena indicate that NNMT might promote anaerobic metabolism [81]. Therefore, more studies are needed in the future to further clarify the roles of NNMT in energy metabolism and the related mechanisms.

NNMT inhibition or *NNMT* knockdown improves insulin sensitivity and normalizes glucose tolerance and fasting blood glucose levels [14], but the mechanisms by which NNMT regulates insulin sensitivity and blood glucose homeostasis are unclear. The liver is the major organ that maintains blood glucose homeostasis in mammals. When blood glucose levels are high, more insulin is excreted from the pancreas, and then, hepatocytes uptake more glucose from the blood to form glycogen under the action of insulin. When blood glucose levels are low, more glucose is produced in the liver by glycogenolysis and gluconeogenesis and then released into the blood. The liver is the organ with the highest level of NNMT expression in mammals. Hong et al. [14] demonstrated that NNMT is a positive modulator in hepatocyte gluconeogenesis but the roles of NNMT in glycogen metabolism in the liver and the mechanisms by which NNMT affects insulin sensitivity have not yet been reported.

## 8. Conclusion

Evidence has shown that NNMT plays important roles in obesity and T2D and that NNMT inhibition significantly increases energy expenditure, reduces body weight and white adipose mass, improves insulin sensitivity, and normalizes glucose tolerance and fasting blood glucose levels. However, the exact mechanisms underlying these phenomena are not yet fully understood. Additionally, trials of oligonucleotide therapeutics and some small-molecule NNMT inhibitors *in vitro* and in preclinical animal models have validated NNMT as a promising therapeutic target to prevent or treat obesity and related T2D, but currently, clinical trials have not yet been reported. Therefore, more researches are still necessary to reveal the roles and action mechanisms of NNMT in obesity and T2D and to develop therapeutics targeting NNMT.
